# Migration Route Out of Africa Unresolved by 225 Egyptian and Ethiopian Whole Genome Sequences

**DOI:** 10.3389/fgene.2016.00098

**Published:** 2016-06-01

**Authors:** Daniel Shriner, Shomarka O. Y. Keita

**Affiliations:** ^1^Center for Research on Genomics and Global Health, National Human Genome Research InstituteBethesda, MD, USA; ^2^Department of Anthropology, Smithsonian InstitutionWashington, DC, USA

**Keywords:** genetic anthropology, Out of Africa, whole genome sequence, ancestry, Y chromosome, mitochondrial DNA (mtDNA), biogeography

Population structure is a fundamental part of population genetics. In coalescent theory, the impact of population structure or a restriction of gene flow is well-studied (Hudson, 1990; Nordborg, 2003). Admixture is inter-mating between previously isolated populations, although the biological characteristics of genetically diverged parental populations can be debated. The pairwise sequentially Markovian coalescent model (Li and Durbin, 2011) and the multiple sequentially Markovian coalescent model (Schiffels and Durbin, 2014), both recently developed methods designed for whole genome sequence analysis, do not model admixture in a formal sense. However, simulations have shown that these models are sensitive to admixture (Li and Durbin, 2011), because admixture increases heterozygosity and consequently appears as an increase in the effective population size. The issue of ancient vs. recent admixture, and the actual time depths, is of concern due to the potentially obscuring effects of a range of evolutionary processes. Both of these models divide time into intervals, theoretically permitting detection of events at different time depths. Consequently, these genetic models have the potential to complement anthropological and archeological studies of the distant past.

Archeological, fossil, and genetic data collectively remain inconclusive regarding the route(s) of modern humans out of Africa: one possible route was north of the Red Sea through Egypt and Sinai and another possible route was south of the Red Sea across the Bab-el-Mandeb Strait (Beyin, 2011). Pagani et al. (2015) described a population genetic study intended to distinguish between these possibilities. The design of their study involved whole genome sequencing of 100 recent Egyptian emigrants living in Lebanon and 25 Amhara, 25 Oromo, 25 Somali, 25 Wolayta, and 25 Gumuz from present-day Ethiopia. The authors used the 1000 Genomes CEU sample as a proxy for “non-African” ancestry and the Gumuz sample as a proxy for “African” ancestry. They then reconstructed the “African” components of the Egyptian and remaining Ethiopian genomes and compared them to the 1000 Genomes YRI, CHB, TSI, and GIH samples. The authors hypothesized that the “African” component of the Egyptian genomes should be more similar to “non-African” genomes under a northern route. Conversely, the “African” component of the Ethiopian genomes should be more similar to “non-African” genomes under a southern route. The authors reported enrichment of the “African” component of the Egyptian genomes, which they interpreted as evidence in favor of the northern route.

The authors' analyses involve two major critical assumptions, one involving population structure and the other involving time. With respect to population structure, the authors assumed that both the Egyptians and Ethiopians could be described using a problematic continental framework, i.e., “African” and “non-African” components which often mask ideas about what constitutes African. Progress has been made: analyses of the genetic structure of autosomal data from global surveys of thousands of individuals have revealed multi-way ancestral compositions at a sub-continental level of resolution, likely reflecting evolution of local or regional populations (Tishkoff et al., 2009; Shriner et al., 2014). Three limitations of these types of studies are (1) the extent to which convenience samples are used, in comparison to a complete catalog of all ethno-linguistic or biogeographical groups (since ethno-linguistic groups have varying time depths), (2) the extent to which populations in such studies are arbitrary constructs (Gannett, 2003), and (3) the appropriateness of divergence by isolation to model the genealogical relationships among ancestries. Given these caveats, the ancestral compositions of samples of modern Egyptians and Ethiopians, as well the reference CEU sample, have been previously estimated (Shriner et al., 2014) and are summarized in Table 1. Notably, the two Egyptian samples we used include a low level of Cushitic ancestry but no Nilo-Saharan ancestry. This absence implies a lack of coverage of the full geographical range of Egyptians, including Nubians who today speak a Nilo-Saharan language (Dobon et al., 2015). There is also no evidence of coverage of individuals representing the Egyptian or Coptic language. Similarly, Figure 1B of Pagani et al. (2015) depicts “East African” ancestry, similar to the ancestry of the Gumuz (who speak a Nilo-Saharan language), constituting < 10% of the Egyptians.

**Table 1:** **Ancestral composition based on autosomal data (Shriner et al., 2014)**.

**Ancestry**	**Egyptian (%)**	**Gumuz (%)**	**Amhara (%)**	**Oromo (%)**	**Wolayta (%)**	**Ethiopian Somali (%)**	**Somali (%)**	**Ari Blacksmith (%)**	**South Sudanese (%)**	**CEU (%)**
Arabian	26.4	0	18.7	10.8	7.3	0	0	0	0	0
Berber	12.6	0	1.6	0	0	0	0	0	0	0
Cushitic	13.8	0	45.0	43.4	37.5	63.2	69.6	0	0	0
Levantine-Caucasian	25.0	0	0	0	0	0	0	0	0	9.4
Niger-Congo	5.4	0	0	0	0	0	0	0	13.8	0
Nilo-Saharan	0	65.0	17.5	23.7	16.6	26.8	30.4	0	86.2	0
Northern European	0	0	0	0	0	0	0	0	0	51.1
Omotic	0	35.0	17.3	22.0	38.7	10.0	0	100	0	0
Southern European	16.7	0	0	0	0	0	0	0	0	39.5

The Egyptian samples were described in Henn et al. (2012) and Behar et al. (2010); the Gumuz, Amhara, Oromo, Wolayta, Ethiopian Somali, Somali, Ari Blacksmith, and South Sudanese samples were described in Behar et al. (2010) and Pagani et al. (2012); and the CEU sample was described by The International HapMap Consortium (2005).

Regardless of the labels given to ancestries, which typically are presumed to be geographically or linguistically based, there are two problems with the data of Pagani et al. (2015). One problem is that their sample of modern Egyptians, like ours, does not reflect all modern Egyptians. Furthermore, it has not been established that original Nile Valley inhabitants are in some sense covered. The genetic compositions of core Afroasiatic (including Egyptian) and Nilo-Saharan speakers are not known fully. The authors chose a subset of five Ethiopian samples from a larger set (Pagani et al., 2012) on the basis of maximizing genetic and cultural diversity. This approach led to a choice of samples all containing substantial ancestral heterogeneity (Table 1), which confounds inference. We believe a better design principle for sample selection is to minimize ancestral heterogeneity, e.g., as used by Tishkoff et al. (2009) in their supervised clustering analysis. Of the Pagani et al. (2012) samples, better choices are Somali, rather than Ethiopian Somali, to represent Cushitic ancestry; Ari Blacksmith, rather than Wolayta, to represent Omotic ancestry; and South Sudanese, rather than Gumuz, to represent Nilo-Saharan ancestry (Table 1). Additionally, samples from Arabian, Levantine, and Maghrebi populations should have been included.

A second problem is that, of all the ancestries present in the Egyptian and Ethiopian samples, ancestry unique to and common in Ethiopians who currently speak an Omotic language is the most divergent (Shriner et al., 2014). Consequently, both “African” and “non-African” genomes are expected *a priori* to be more similar to the “African” component of Egyptian genomes than the “African” component of Ethiopian genomes, solely on the basis of genetic distance and independent of genealogical relationships among ancestries. To see this, suppose that East African ancestry in the Egyptians and Ethiopians is identical. Then, comparison of “non-Africans” to this East African component will be inconclusive. On the other hand, suppose that East African ancestry is a combination of Nilo-Saharan and Cushitic ancestries in the Egyptians with an additional Omotic contribution in the Ethiopians (Pagani et al., 2012). Then, given that Omotic ancestry is essentially restricted to Ethiopia, “non-Africans” will be more similar to Egyptians' East African than Ethiopians' East African.

With respect to time, the authors assume that “modern African populations are representative of those at the time of the exit” (Pagani et al., 2015). This assumption may be problematic because of underlying typological assumptions that include conceptualizing and treating geographically or linguistically defined populations such that the same genetic patterns would manifest in any sample from the geographical range or branch of the language family across time. More directly, it would have been useful if the authors had estimated the split time between the African components of the modern Egyptian and Ethiopian genomes. If this split time postdates Out of Africa, then we may infer that the African ancestors of the modern Egyptians and Ethiopians were not genetically differentiated at the time of exit and therefore that a northern route and a southern route are indistinguishable.

There are five lines of evidence against the assumption of representativeness. One, the authors assessed the split times of the “African” components of the modern Egyptian and Ethiopian genomes compared to a “non-African” CEU genome. Despite overlapping time intervals and a lack of formal statistical assessment, the authors inferred a higher similarity between “non-African” and Egyptian “African” components; we find the results to be inconclusive. Two, the authors assessed the split times of the “African” components of the modern Egyptian and Ethiopian genomes compared to a West African YRI genome and an East African Gumuz genome. The split times compared to the YRI genome were 21,000 and 37,000 years ago for Egyptians and Ethiopians, respectively, and even more recent compared to the Gumuz genome. Thus, the African ancestors of the West African YRI, the East African Gumuz, and the “African” components of the modern Egyptian and Ethiopian genomes had not split at the time of exit. Three, reconstruction of the phylogenetic history of autosomal ancestries showed that none of the autosomal ancestries of modern Egyptians and modern Ethiopians had yet diverged at the time of exit (Shriner et al., 2014). Four, in the authors' Supplement, the “African” component includes Y haplogroups A3b2, B2, and E, whereas the “non-African” component includes descendants of Y haplogroup F (specifically G, J, L, R, and T), which is not descended from A3b2, B2, or E. Five, also in the authors' Supplement, the mitochondrial DNA haplogroup L3, the ancestor of M and N haplogroups, is present in both modern Egyptians and modern Ethiopians. Thus, both Y and mitochondrial DNA are inconclusive.

Ancient DNA might help to resolve the question of the route out of Africa, if temporally appropriate specimens can be found. The individual named *Bayira* discovered in the Mota Cave in Ethiopia dated to ~4500 years ago (Gallego Llorente et al., 2015), which is not old enough. Also, *Bayira* was determined to be ancestrally homogeneous for Omotic ancestry (Gallego Llorente et al., 2015). By comparison, our data set contains the equivalent of 69 individuals ancestrally homogeneous for Omotic ancestry (Shriner et al., 2014), reflecting the ability of ancestry analysis to disentangle recent admixture.

Taken together, the autosomal, Y chromosome, and mitochondrial DNA data support the conclusion that the indigenous African components of the specific samples of modern Egyptians and modern Ethiopians studied by Pagani et al. (2015) are uninformative with respect to the origin of non-Africans. The available data suggest that the separation of ancient Egyptians and ancient Ethiopians postdates Out-of-Africa. In the absence of ancient DNA specimens, estimation of genetic profiles of core Afroasiatic and Nilo-Saharan speakers requires phylogenetic techniques to reconstruct ancestral states.

## Author contributions

All authors listed, have made substantial, direct and intellectual contribution to the work, and approved it for publication.

## Funding

This research was supported by the Intramural Research Program of the Center for Research on Genomics and Global Health (CRGGH). The CRGGH is supported by the National Human Genome Research Institute, the National Institute of Diabetes and Digestive and Kidney Diseases, the Center for Information Technology, and the Office of the Director at the National Institutes of Health (Z01HG200362).

### Conflict of interest statement

The authors declare that the research was conducted in the absence of any commercial or financial relationships that could be construed as a potential conflict of interest.

## References

[B1] Behar D. M. Yunusbayev B. Metspalu M. Metspalu E. Rosset S. Parik J.. (2010). The genome-wide structure of the Jewish people. Nature 466, 238–242. 10.1038/nature09103 20531471

[B2] Beyin A. (2011). Upper Pleistocene Human Dispersals out of Africa: a review of the current state of the debate. Int. J. Evol. Biol. 2011:615094. 10.4061/2011/615094 21716744 PMC3119552

[B3] Dobon B. Hassan H. Y. Laayouni H. Luisi P. Ricaño-Ponce I. Zhernakova A.. (2015). The genetics of East African populations: a Nilo-Saharan component in the African genetic landscape. Sci. Rep. 5:9996. 10.1038/srep09996 26017457 PMC4446898

[B4] Gallego Llorente M. Jones E. R. Eriksson A. Siska V. Arthur K. W. Arthur J. W.. (2015). Ancient Ethiopian genome reveals extensive Eurasian admixture throughout the African continent. Science 350, 820–822. 10.1126/science.aad2879 26449472

[B5] Gannett L. (2003). Making Populations: bounding Genes in Space and in Time. Philos. Sci. 70, 989–1001. 10.1086/377383

[B6] Henn B. M. Botigué L. R. Gravel S. Wang W. Brisbin A. Byrnes J. K.. (2012). Genomic ancestry of North Africans supports back-to-Africa migrations. PLoS Genet. 8:e1002397. 10.1371/journal.pgen.1002397 22253600 PMC3257290

[B7] Hudson R. R. (1990). Gene genealogies and the coalescent process, in Oxford Surveys in Evolutionary Biology, eds Futuyma D. Antonovics J. (New York, NY: Oxford University Press), 1–44.

[B8] Li H. Durbin R. (2011). Inference of human population history from individual whole-genome sequences. Nature 475, 493–496. 10.1038/nature10231 21753753 PMC3154645

[B9] Nordborg M. (2003). Coalescent Theory, in Handbook of Statistical Genetics, 2nd Edn, eds Balding D. J. Bishop M. Cannings C. (Hoboken, NJ: John Wiley & Sons, Ltd.), 602–635.

[B10] Pagani L. Kivisild T. Tarekegn A. Ekong R. Plaster C. Gallego Romero I.. (2012). Ethiopian genetic diversity reveals linguistic stratification and complex influences on the Ethiopian gene pool. Am. J. Hum. Genet. 91, 83–96. 10.1016/j.ajhg.2012.05.015 22726845 PMC3397267

[B11] Pagani L. Schiffels S. Gurdasani D. Danecek P. Scally A. Chen Y.. (2015). Tracing the route of modern humans out of Africa by using 225 human genome sequences from Ethiopians and Egyptians. Am. J. Hum. Genet. 96, 986–991. 10.1016/j.ajhg.2015.04.019 26027499 PMC4457944

[B12] Schiffels S. Durbin R. (2014). Inferring human population size and separation history from multiple genome sequences. Nat. Genet. 46, 919–925. 10.1038/ng.3015 24952747 PMC4116295

[B13] Shriner D. Tekola-Ayele F. Adeyemo A. Rotimi C. N. (2014). Genome-wide genotype and sequence-based reconstruction of the 140,000 year history of modern human ancestry. Sci. Rep. 4:6055. 10.1038/srep06055 25116736 PMC4131216

[B14] The International HapMap Consortium (2005). A haplotype map of the human genome. Nature 437, 1299–1320. 10.1038/nature04226 16255080 PMC1880871

[B15] Tishkoff S. A. Reed F. A. Friedlaender F. R. Ehret C. Ranciaro A. Froment A.. (2009). The genetic structure and history of Africans and African Americans. Science 324, 1035–1044. 10.1126/science.1172257 19407144 PMC2947357

